# Mitigation strategies to reduce particulate matter concentrations in civil engineering laboratories

**DOI:** 10.1007/s11356-024-31926-w

**Published:** 2024-01-17

**Authors:** Irem Bayram Zumrut, Ozge Akboga Kale, Yilmaz Ogunc Tetik, Selim Baradan

**Affiliations:** 1 Department of Civil Engineering, Ege University, Izmir, Turkey; 2 Department of Civil Engineering, Izmir Demokrasi University, Izmir, Turkey; 3 Department of Civil Engineering, Mugla Sitki Kocman University, Mugla, Turkey

**Keywords:** Particulate matter, Respirable dust, Civil engineering, Indoor air quality, Air pollution, Hazard control, Laboratory

## Abstract

In the departments of civil engineering, many experiments are conducted in laboratories for educational and research purposes. Varying degrees of respirable dust are generated as the outcome of these experiments, which could cause harm to instructors’ and students’ health. This study is devised to highlight the importance of indoor air quality in university laboratories. As part of the research, four different particulate matter (PM) sizes (PM_1.0_, PM_2.5_, PM_4.0_, and PM_10_) were measured during specific experiments—sieve analysis, preparation of the concrete mixture, crushing aggregate by jaw crusher, dynamic triaxial compression test, sieve analysis of silt specimen, cleaning sieve by an air compressor, and proctor compaction test—being conducted periodically in the laboratories of civil engineering departments. The measured values are mainly high compared to indoor air quality standards. Mitigation strategies were applied to reduce indoor air PM levels in the three experiments that contained the highest PM levels. The results have shown that mitigation strategies applied as control measures could make a remarkable difference in protecting instructors and civil engineering students.

## Introduction

With the increase in the world’s total population, environmental problems, including air quality in indoor and outdoor environments, are rising gradually. Air pollutants in ambient air have adverse impacts on the respiratory and cardiovascular health of the population (Weichenthal et al. 2013). As an environmental health risk, the indoor and outdoor air quality parameters need to be monitored regularly. In today’s cities, the need for indoor air quality measurements has increased as more people spend a significant part of their daily lives in indoor environments (Keskin and Dilmac 2017).

Construction activities are one of the major sources of environmental pollution and human health threats (Li, et al. 2019). Construction activities are more hazardous than other production processes due to their production rates of waste, and this causes several public health problems (Holton et al. 2008). As 66% of the earth’s population will be moving toward nonrural areas by 2050, the cities’ activity rate of construction work will increase accordingly (Oliveira et al. 2019). Particulate matter (PM), which is defined as solid or liquid particles suspended in air, occurs from the mechanical processes of materials such as rocks and sand in construction sites (Chang, et al. 2014). PM exposure consisting of construction dust can cause heart and lung diseases, eye irritation, respiratory problems, etc. (Ahmed and Arocho 2019; Sánchez-Soberón, et al. 2019). PM exposure causes approximately 800,000 premature deaths around the world annually (Anderson et al. 2012). Studies show that PM exposure is associated with respiratory diseases (McConnell, et al. 1999; Grigg 2009; Peng et al. 2009; Khoza et al. 2012; Kesavachandran, et al. 2015; Muneku and Naidoo 2015; Zhou, et al. 2019; Yu et al. 2019). In addition to respiratory diseases, studies reveal that the respirable dust could cause cardiovascular and cerebrovascular diseases (Samet et al. 2000; Pope and Dockery 2012), heart disease (Merefield 2002; Haynes 2006), and skin allergies (Ilicin et al. 2005). A study highlighted that exposure to cement dust is a major cause of cancer among construction workers (Peters et al. 2009). Construction dust and its influence on the local environment have become the main concern of the parties, including workers, clients, contractors, and the public (Yuan et al. 2013).

Many researchers have performed studies on construction dust and the measurement of PM concentrations at construction sites. The most measured PM sizes are PM_10_—particles that have a diameter of 10 μm or less—PM_2.5_—particles that have a diameter of 2.5 μm or less—and PM_1.0_—particles that have a diameter of 1.0 μm or less (Zhao et al. 2007). Yan et al. (2018) measured the PM_10_ concentration of a construction site and revealed that it outstripped the local standard of China. Another study demonstrated that the locations near the construction sites are exposed to high concentrations of PM because of construction activities (Haynes and Savage 2007). Azarmi et al. (2014) analyzed the exposure level of coarse particles (PM_2.5–10_), fine particles (PM_2.5_), and ultrafine particles (UFPs) (PM_1.0_) during concrete-related operations. The average PM_10_, PM_2.5_, and PM_1.0_ were calculated, respectively, as 2.82, 1.19, and 0.80 mg/m^3^ for concrete drilling, 1.89, 0.78, and 0.56 mg/m^3^ for concrete mixing, and 3.77, 1.34, and 0.86 mg/m^3^ for concrete cutting. In the literature, there are many studies conducted on construction sites that show similar results (Paschalidou et al. 2016; Tong et al. 2018; Li et al. 2018; Tian et al. 2019; Cheriyan and Choi 2020).

In contrast to open construction sites, laboratory environments differ significantly in characteristics and dust exposure implications. Laboratories are generally controlled, enclosed settings with regulated ventilation, specialized equipment, and personal protective measures (Yang et al. 2019; Bai et al. 2022). Construction sites are dynamic, outdoor areas with limited control over ventilation and a variety of materials (Guo et al. 2022; Chenari et al. 2016). The dispersed nature of construction activities and exposure to natural elements make dust management more challenging. The particle size, occupational health measures, and overall working conditions vary between these environments, emphasizing the importance of tailored safety practices in each setting. The severity of dust hazards in civil engineering laboratory buildings is a multifaceted issue influenced by several critical factors. These include the nature of experiments involving cutting, grinding, and manipulating construction materials, leading to the generation of diverse dust particles (Li et al. 2019). The types of materials used, such as concrete and metals, contribute to the composition and toxicity of the dust (Kunal et al. 2012). Particularly in civil engineering education, laboratory personnel—technicians, specialists, and teaching/research assistants—and students are exposed to dust in the course of experimentation, which could cause adverse health effects. Nevertheless, there are a few studies that concern the university laboratories of other engineering departments (Rumchev et al. 2003; Valavanidis and Vatista 2006; Ugranli et al. 2015). The results showed that PM_10_ and PM_2.5_ levels were lower than their respective limits in the university laboratories.

The aim of the study is novel due to measuring PM concentrations in civil engineering laboratories. The measured experiments are sieve analysis, preparation of the concrete mixture, crushing aggregate by jaw crusher, dynamic triaxial compression test, sieve analysis of silt specimen, cleaning sieve by air compressor, and proctor compaction test conducted periodically in the laboratories of Ege University Civil Engineering Department. Four different PM sizes—PM_1.0_, PM_2.5_, PM_4.0_, and PM_10_—are measured and compared to the indoor air quality standards of the Environmental Protection Agency (EPA), the World Health Organization (WHO), the American Society of Heating, Refrigerating and Air-Conditioning Engineers (ASHRAE), and the occupational health and safety standards of the Occupational Safety and Health Administration (OSHA). The experiments conducted in civil engineering laboratories were characterized by their PM concentrations. Correspondingly, recommendations are suggested to reduce indoor PM levels in civil engineering laboratories, and mitigation strategies are applied to the experiments that have the highest PM concentrations.

## Materials and methods

### Site description

**Fig. 1:**
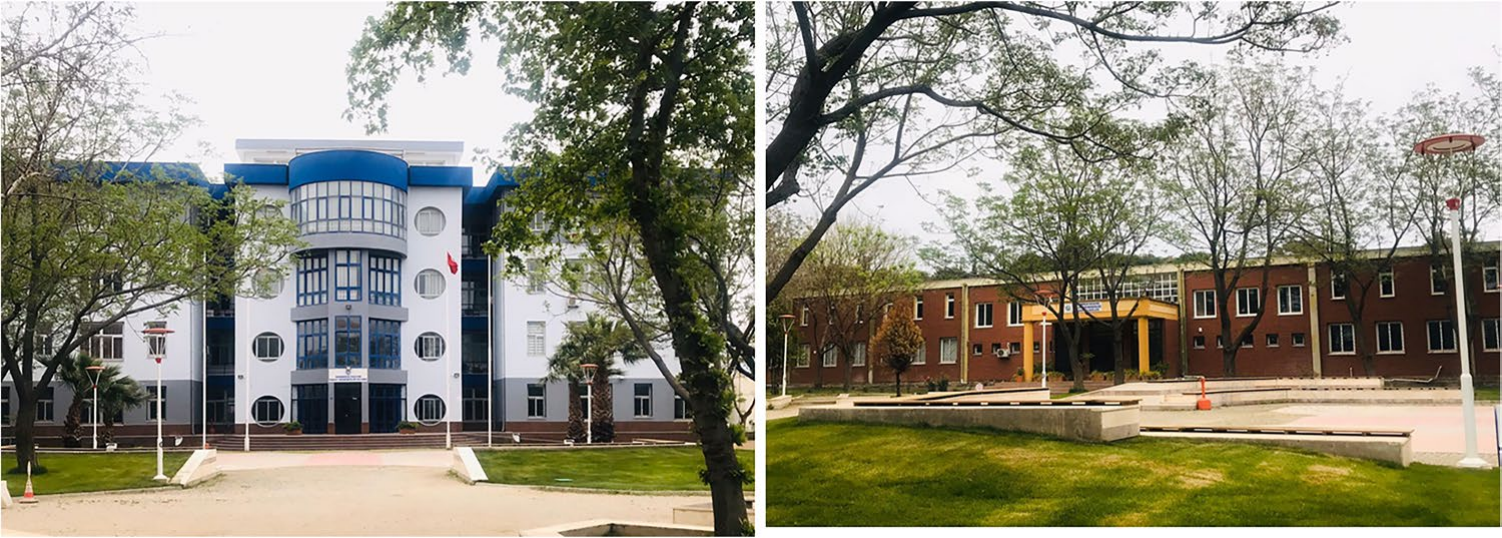
Department of Civil Engineering (left) and Laboratory building (right)

The PM measurements are performed in the laboratory of the Civil Engineering Department of Ege University, located in Izmir province, Turkey. Also, Izmir is a city that has a Mediterranean climate with long, hot, and dry summers as well as mild and rainy winters. The total precipitation in Izmir averages 689 mm annually. The average annual temperature is 17.9 °C; the maximum temperature during winter varies usually between 10 and 16 °C, while it ranges between 30 and 36 °C during summer. The average wind speed is 3.0 m/s (General Directorate of Meteorology 2019). The Civil Engineering Laboratory building of Ege University is located near the main building of the Civil Engineering Department in İzmir, Turkey (Fig. 1).

The laboratory complex of the civil engineering department consists of four different divisions in a single building, such as geotechnics, construction materials, transportation, and structural engineering. The physical conditions are similar in all parts of the laboratory. All the laboratories have an inherently dusty environment due to the storage of materials such as cement, aggregate, and mineral additives in the same area. Wet cleaning methods are not preferred to keep the stored materials as dry as possible. The building is away from traffic-related pollutants. Although the laboratories have air conditioning units, they are usually ventilated naturally via doors and windows. Despite having similar physical conditions in each laboratory, used materials, devices, and experiments differ from each other.

In an academic year, an average of 40 students are exposed to dust daily in the Ege University Civil Engineering Laboratories. Due to courses and lessons progressing, experiments are conducted continuously. Since there are instructors and technicians who have an office in the laboratory, the exposure of those who permanently reside there is high.

### Data collection

Six experiments are selected out of more than 30 experiments regularly conducted in civil engineering laboratories. The selection decision is based on their emission potential; therefore, some common experiments in different civil engineering divisions are excluded from the scope of this study due to their potentially low dust emissions. Emission potential refers to the capacity of a substance, typically a pollutant or hazardous material, to be released into the environment under specific conditions. Construction materials and geotechnics laboratories are selected for PM measurements. Selecting appropriate experiments is a critical aspect of scientific research and experimentation. The principles of experiment selection involve strategic decision-making to ensure the experiments conducted are relevant and valid and contribute meaningfully to the research objectives. Key principles include the frequency of experiments, type of materials used, and dust generation potential (Table 1). The frequency of experiments conducted in a laboratory setting significantly impacts the potential for dust formation. Particularly, operations involving cutting, grinding, or construction materials may intensify dust generation, subsequently elevating potential risks within the laboratory environment. Moreover, the types of materials employed, especially construction materials like concrete, exert a determinant influence on the quantity and characteristics of the generated dust.

**Table 1:** Measured experiments and the locations

Division	Location	Experiment
Construction materials	ML-1	1: Sieve analysis
	ML-2	2: Preparation of concrete mixture
	ML-3	3: Crushing aggregate by jaw crusher
Geotechnics	GL-1	4: Dynamic triaxial compression test
		5: Sieve analysis of the silt specimen
		6: Cleaning sieve by air compressor
	GL-2	7: Proctor compaction test

**Fig. 2:**
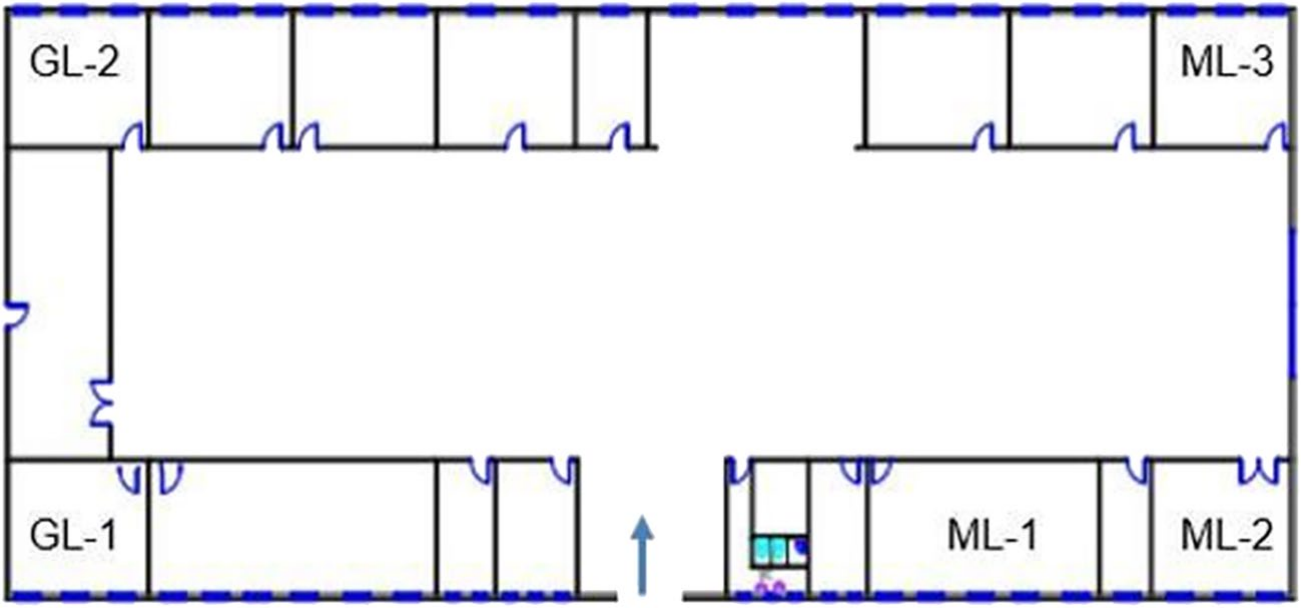
Floor plan of the laboratory building

Three commonly performed experiments in materials in construction laboratories (ML)—sieve analysis in ML-1, preparation of concrete mixture in ML-2, and crushing aggregate by jaw crusher ML-3—are selected for dust measurement. Four experiments from geotechnics laboratories (GL)—preparation of the silt specimen by using a plastic knob for a dynamic triaxial compression test, sieve analysis of the silt specimen and cleaning sieve by air compressor in GL-1, and proctor compaction test in GL-2—are selected. All experiments were enumerated in the floor plan of the laboratories, and the location of the measurements is shown in Fig. 2.

As evident from Fig. 2, each of the four laboratories (GL-1, GL-2, ML-2, and ML-3) holds an equal floor area of 35 m^2^. All four laboratories are equipped with three windows each. However, the ML-1 laboratory spans an area of 55 m^2^ and accommodates five windows. All window dimensions remain uniform, with each window having an area of 2 m^2^. These windows are capable of opening on a single side, allowing for an ingress area of 0.75 m^2^ when opened for ventilation. There is no air conditioning system in any of the laboratories; only natural ventilation is available. Throughout the sampling procedure, all windows were kept closed during experiments to maintain consistency and enable comparative measurements.

Experiments in laboratories comprise different variable particle sizes based on the materials used. The first measurement in the material laboratory was performed during the sieve analysis. Sieve analysis—also called grain size analysis—is usually performed for the classification of soils by separating the granular material using various kinds of sieves to obtain grain size distributions. The materials are placed on the sieve set and shaken to separate the granular materials. Since there is no mechanical sieve shaker in the laboratory, the sieve analyses are performed manually. During the shaking process, a huge amount of dust emanates from the materials. Testers are subjected to extreme dust exposure during this process. The test is performed in ML-1, where the ambient condition is dusty due to the stored materials in this area. Moreover, particles could easily be suspended in the air due to the lack of wet cleaning methods. The typical duration for conducting a sieve analysis is usually 20 min. During the sampling process, the experiments were repeated 3 times. To ensure consistency across all experiments, the duration of each experiment was extended to at least 1 h.

Another commonly conducted experiment is the preparation of the concrete mixture. Aggregate, water, cement, and other chemical additions are mixed in the mixer and placed into cubic molds. Researchers prepare various concrete mixtures in the materials of construction laboratories and test their compressive performance to decrease ingredients like cement. Dust usually occurs during the mixing process. The measurements were sampled during the preparation of a lightweight concrete mixture in ML-2. The preparation of this concrete mixture took approximately 30 min. Measurements were taken on three separate occasions. In ML-3, the experiments involving crushing aggregate using a jaw crusher were conducted within a specified duration of 20 min and repeated three times for measurement consistency. The purpose of the jaw crusher is to reduce the rocks, materials, or recycling products to smaller sizes for the next crushing stages. During the crushing process, occasional placement of the materials is required, and that is when dust emanates.

In geotechnical laboratories, there are also many experiments and tests being conducted due to soil behavior analysis. A dynamic compression test is a common method to measure the dynamic properties and liquefaction resistance of soils. The sample is placed on a pedestal, and dynamic loading is applied by the load cell while confining pressure is also applied by the water cell. During the experiment, the soil specimen is under water pressure, which prevents dust generation. However, dust could be produced during the specimen preparation, and the measurements are performed in this stage. The duration of the experiment was approximately 40 min, and the process was repeated 3 times. Other measurements were taken during the sieve analysis of the specimens and cleaning of the sieve by the air compressor. The process of sieve analysis is similar to that in construction material laboratories. Nevertheless, owing to the material being silt, the duration of the sieve analysis experiment exceeded that of the previous one which lasted 30 min and was repeated 3 times. The used sieves are cleaned by the air compressor, and this process could cause massive amounts of dust concentration. The process was notably brief, lasting approximately 5 min, and it was repeated at least 10 times to ensure thoroughness and reliability in the measurement. One of the most commonly performed experiments in the geotechnical laboratory is the proctor compaction test. The test aims to determine the optimal moisture content of soil corresponding to maximum dry density. Soil is placed in a cylindrical mold and compacted in three layers using a hammer to provide standard proctor energy. The process was repeated with five different moisture contents, which lasted more than an hour.

### Sampling equipment

**Fig. 3:**
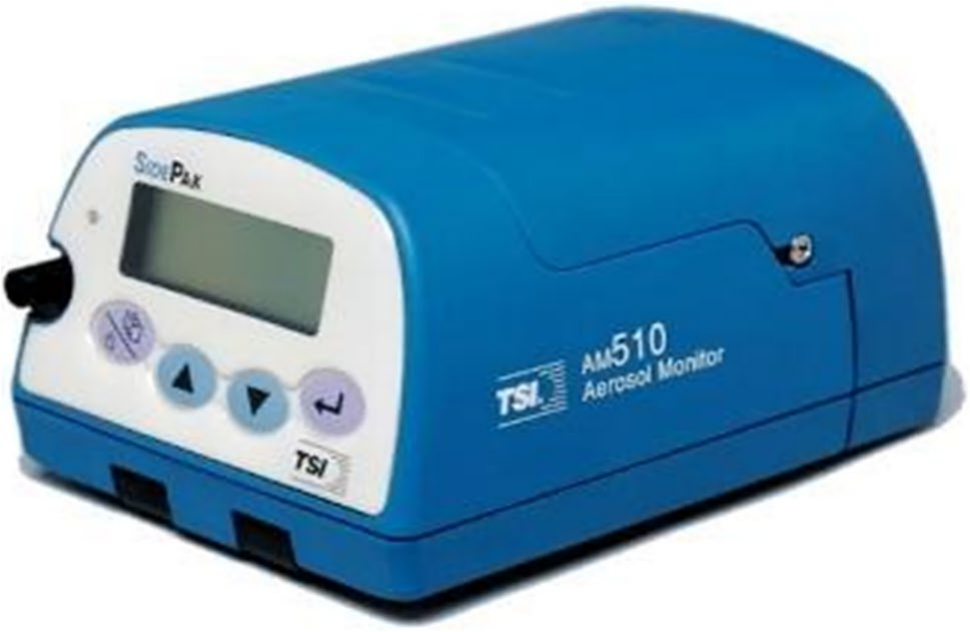
TSI SidePak Aerosol Monitor

PM concentrations were sampled by using the TSI SidePak Aerosol Monitor (AM510). The device uses a light scattering method to determine the mass concentration of particles in real-time (Fig. 3). The device consists of four different inlets of PMs—PM_1.0_, PM_2.5_, PM_4.0_, and PM_10_. The sampler device is calibrated according to the standard ISO 12103–1, A1 test dust (Arizona Test Dust) (TSI, 2012). The aerosol monitor complies with the PD CEN/TR 16013–3:2012 Standard. The sampler is located approximately 1–1.5 m away from the executed area of the experiment.

## Results and discussion

This study reports the students’ and laboratory instructors’ PM exposure concentrations during different civil engineering experiments. All PM exposure measurements were conducted in the 2019–2020 academic year. Every experiment was sampled three different times and repeated during the working hours of the day. The duration of sampling for all experiments is equal to an hour. Zero calibration was applied before every use. The same procedure was followed in all experiments to keep the data collection method consistent. To generate a baseline for the measurements, each laboratory environment was measured without performing any experiments. It was ensured that no other operations were present while measuring the environment. Similar results are achieved in both laboratories for all PM rates, and the concentration rates are given in Table 2.

**Table 2:** Baseline PM values of laboratories (without any experiments)

Laboratories	PM_1.0_	PM_2.5_	PM_4.0_	PM_10_
Construction materials				
ML-1	28	31	33	34
ML-2	27	30	33	34
ML-3	28	30	34	35
Geotechnics				
GL-1	28	30	30	33
GL-2	31	32	33	34
Average (µg/m^3^)	28.4	30.6	32.6	34.0

The PM concentration rates were measured for each experiment and given in Table 3. Each experiment was sampled three times for four different PM sizes. Values—minimum, maximum, and averages—in the table are means of these three measurements. The experiments are coded from 1 to 7 based on the following order: sieve analysis, preparation of the concrete mixture, crushing aggregate by jaw crusher, dynamic triaxial compression test, sieve analysis of the silt specimen, cleaning sieve by air compressor, and proctor compaction test (also listed in Table 1).

**Table 3:** PM concentrations of measured experiments

Experiments	PM_1_ (µg/m^3^)			PM_2.5_ (µg/m^3^)			PM_4.0_ (µg/m^3^)			PM_10_ (µg/m^3^)		
	*min*	*max*	*mean*	*min*	*max*	*mean*	*min*	*max*	*mean*	*min*	*max*	*mean*
1	29	3492	474.3	10	5506	587	26	7903	972.3	21	16,966	1463
2	23	13,814	273.3	56	3129	312.3	99	5986	582.7	200	11,022	781.7
3	12	1810	250.3	10	3516	448.7	43	4171	555	91	5645	639.7
4	11	816	83.3	41	519	152.3	12	1298	183.7	104	1357	306.3
5	16	5158	127	36	1616	144.7	79	1058	180	26	5096	276
6	8	1717	151	43	2187	292.3	72	3109	591.7	114	16,243	1181
7	42	976	151	56	2190	173	19	2265	186.3	55	1853	216

For PM_1.0_ emissions, the highest recorded value is for sieve analysis in the construction material laboratory (474.3 µg/m^3^), and the lowest value is for the preparation of the specimen on a dynamic triaxial compression test (83.3 µg/m^3^). For PM_2.5_, the highest recorded value is for sieve analysis in the construction material laboratory (587 µg/m^3^), and the lowest value is for sieve analysis of the silt specimen (144.67 µg/m^3^). For PM_4.0_, the highest recorded value is again for sieve analysis in the construction material laboratory (972.3 µg/m^3^), and the lowest value is for sieve analysis of the silt specimen (180 µg/m^3^). For PM_10_, the highest recorded value is for sieve analysis in the construction material laboratory (1463 µg/m^3^), and the lowest value is for the proctor compression test (216 µg/m^3^). As observed from the PM results, the maximum concentrations in every PM size were detected during the sieve analysis experiment in the construction material laboratory.

**Fig. 4:**
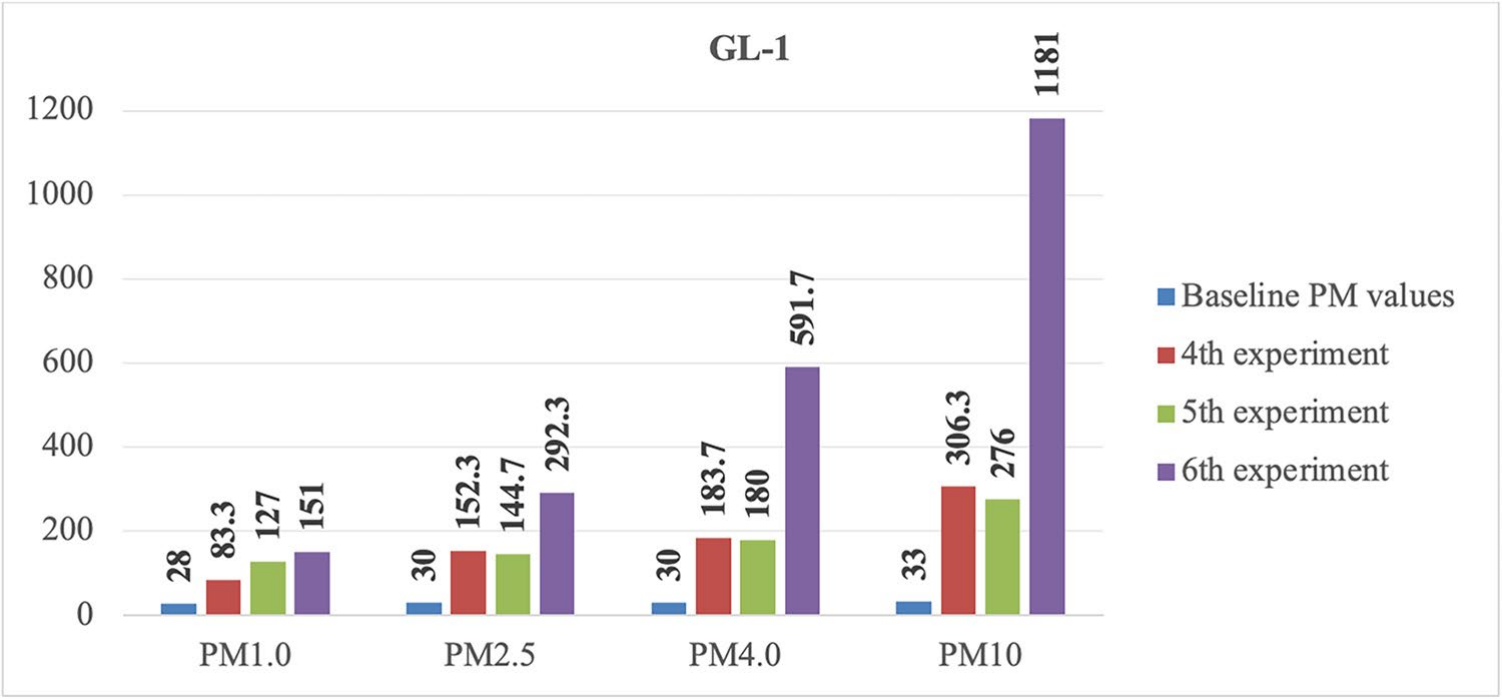
The increments of PM levels in the GL-1 laboratory

In order to indicate the post-experiment increase in PM levels, the baseline values of each laboratory and the PM levels during the experiments were compared. For effective illustration and discussion of the rise in PM levels, the graph representing the GL-1 laboratory has been specifically selected regarding the other laboratories—ML-1, ML-2, ML-3, and GL-2—which have only conducted one experiment. In contrast, the GL-1 laboratory has undergone three distinct experiments (Fig. 4). The comparison between baseline values and subsequent experiments highlighted not just the incremental changes but also provided a basis for assessing the consistency or variability of PM level alterations in different experimental conditions. This approach enabled a more nuanced evaluation of the experimental impact on PM levels, offering valuable insights into potential trends and implications for further investigation.

The PM concentrations are compared to the indoor air quality (IAQ) standards provided by the WHO (air quality guidelines: 25 and 50 μg/m^3^ for PM_2.5_ and PM_10_, respectively), EPA (35 and 150 μg/m^3^ for PM_2.5_ and PM_10_, respectively), and ASHRAE (15 μg/m^3^ for PM_2.5_) (WHO 2005; EPA 2012; ASHRAE 2013). Notably, the WHO limits are primarily defined for ambient air, but they could be applied to indoor environments. The measured PM concentrations of all experiments are above the limits of air quality standards. Also, the PM concentration level for each experiment was compared to the occupational health and safety standard to assess the health hazards of the instructors and students exposed to the particulate matter. OSHA defines the PM exposure limits as 5000 μg/m^3^ for PM_2.5_ and 15,000 μg/m^3^ for PM_10_ (OSHA 2011). The mean values of the experiments are below the threshold values; however, the maximum values show that PM exposure could be hazardous for the health of students and instructors during the sieve analysis and cleaning of the sieve by the air compressor.

When measured PM concentrations (results for PM_2.5_ and PM_10_ scale are between 173 and 587 µg/m^3^ and 216 and 1463 µg/m^3^, respectively) were compared with similar studies, they were found to be much higher than the other engineering laboratories (Ugranli et al. 2015; Rumchev et al. 2003; Valavanidis and Vatista 2006). The primary reasons for this difference lie in the characteristics of materials used, procedures employed during experiments, and the laboratory conditions specific to the civil engineering department. The most significant contrast between civil engineering laboratories and their engineering counterparts resides in the materials utilized during experiments. Particularly in geotechnical and structural material divisions, materials such as sand, gravel, clay, silt, and similar substances possess a substantial potential for generating dust. These materials undergo processes like compression, crushing, and mixing, among others, leading them to transform into airborne particles. Another notable factor is the laboratory conditions under which PM concentration is measured. Laboratories lack dedicated air conditioning systems and rely solely on natural ventilation by opening windows. During the measurements, the windows were not opened due to prevailing weather conditions, causing the PM values to exceed those of other controlled engineering laboratories.

PM concentration values were also compared with values from construction sites. Ahmed and Arocho (2019) measured the PM concentrations (PM_1.0_, PM_2.5_, PM_4_.0, and PM_10_) at different construction sites. The average PM values varied between 6.61 and 29.65 µg/m^3^ for PM_1.0_, 7.17 and 18.63 µg/m^3^ for PM_2.5_, 9.05 and 30.44 µg/m^3^ for PM_4.0_, and 11.39 and 25.05 µg/m^3^ for PM_10_. These values are lower than the civil engineering laboratories’ PM concentrations. The difference between the two studies for PM concentrations could be associated with the measured activity and the sampling duration. In this study, measurements were taken during the experiment, but before and after activities were not included. In addition, the weather data were not recorded during the measurements due to the fact that there is no correlation in the literature on PM emission level with temperature, humidity, wind speed, etc. (Ahmed and Arocho 2019).

Studies related to particulate matter air pollution mostly focused on the emission of PM_2.5_ and PM_10_. Those two sizes also underlie all the standards. However, this study considered two more particulate matters, having different PM sizes—PM_1.0_ and PM_4.0_—for additional emission information from the university laboratory. The measurement of these particle sizes allowed a more detailed examination of particles of varying sizes present in laboratory environments. Smaller particles like PM_1.0_ have a deeper impact on the performance of the lungs and the total respiratory system than PM_2.5_ (Yang et al. 2020; Hu et al. 2022). Toxicological evidence indicates that smaller PM_1.0_ particles pose a greater hazard in terms of cytotoxic effects and inflammation compared to PM_2.5_ particles (Jalava et al. 2015). Thus, measuring PM_1.0_ particles offered a more specific evaluation concerning the respiratory health of laboratory personnel and construction workers. Conversely, larger particles—PM_4.0_—held significance in understanding the effects on indoor air quality (Scheepers et al. 2017). Measuring PM_4.0_ particles enabled a more comprehensive understanding of the distribution and impact of various particles generated from the processing of different materials or during experimental procedures. These additional measurements provided a more in-depth insight into particle concentration within laboratory environments or construction processes, supplementing the standard PM_2.5_ and PM_10_ measurements.

## Results of applied mitigation strategies

**Fig. 5:**
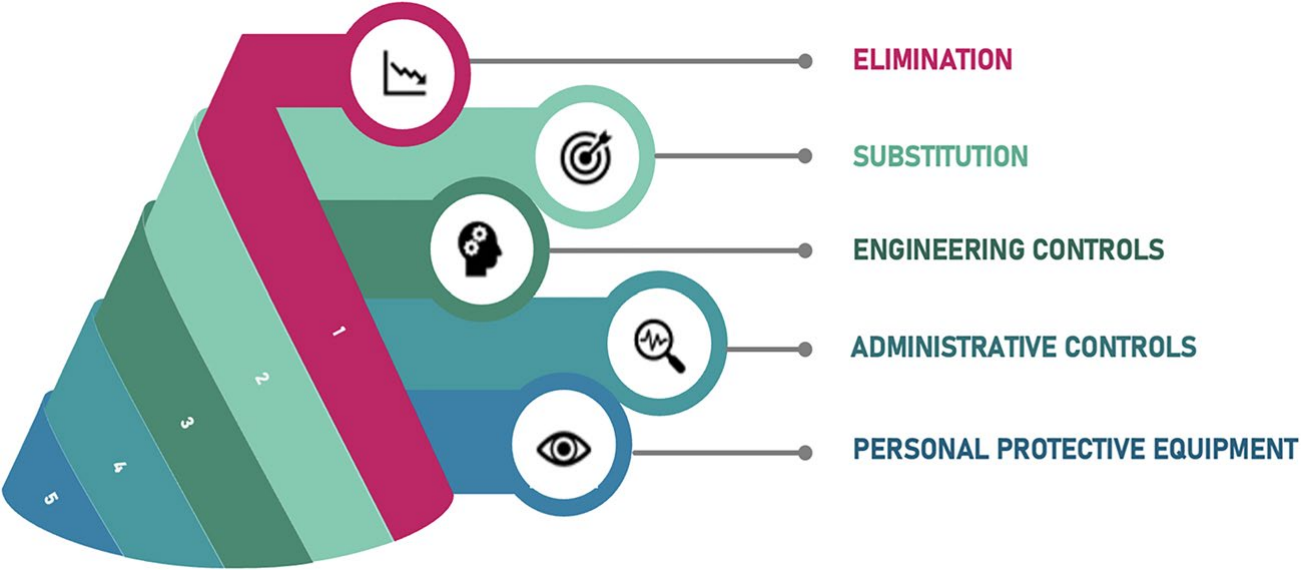
Hierarchy of controls to prevent risk-related hazards

The findings of the study indicated that civil engineering laboratories are responsible for high levels of PM concentrations. In terms of IAQ conditions in laboratories, workplace conditions pose a risk to students and instructors for respiratory tract diseases such as lung cancer, asthma, silicosis, and chronic obstructive pulmonary disease when considering the materials used. Preventive measures should be applied to prevent health risks according to the risk hierarchy principles that are presented in Fig. 5 (NIOSH 2017).

The first step in the hierarchy of control is elimination, which means physically removing hazards. This step could not be applied due to the continuity of civil engineering education and services provided to the AEC industry. The second step is substitution—the second most effective hazard control principle—which involves replacing the equipment causing hazards with other equipment or machinery that does not cause hazards. This step was applied to experiments that constitute high risks, such as sieve analysis, preparation of the concrete mixture, and crushing aggregate by jaw crusher. For sieve analysis, an industrial vibrating sieve machine was provided to vibrate automatically in closed canisters. Also, industrial closed concrete mixers and crushers were obtained for the testing of particulate matter generation. By implementing the substitution strategy as per the hierarchy of controls, we observed considerable reductions in PM concentrations during sieve analysis (1), concrete mixture preparation (2), and crushing aggregate using a jaw crusher (3) (Table 4). Remarkably, these reduced PM concentrations fell below the limit values specified by the air quality standard of the EPA. This correlation between the deliberate application of substitution strategies and the subsequent reduction in PM levels highlights the direct impact of the chosen hazard control approach on experimental outcomes. It emphasizes the profound significance of seemingly minor alterations in equipment or methodology in protecting the health and safety of the individuals involved.

**Table 4:** PM concentrations of substitution measures applied to experiments

Experiments	PM_1.0_ (µg/m^3^)			PM_2.5_ (µg/m^3^)			PM_4.0_ (µg/m^3^)			PM_10_ (µg/m^3^)		
	*min*	*max*	*mean*	*min*	*max*	*mean*	*min*	*max*	*mean*	*min*	*max*	*mean*
1	15	506	21.1	10	1016	33.2	23	1233	85.9	17	1985	149.4
2	13	733	11.2	56	867	14.3	56	916	71.0	42	1664	133.7
3	8	666	18.7	10	542	28.6	12	714	62.8	30	978	111.6

The third step is called engineering control; it does not eliminate hazards but rather isolates personnel and students from hazards. The principle is to separate personnel from dust by using a general or local exhaust ventilation system to remove the dusty air. Local exhaust ventilation is a common and useful strategy described as the extraction of the air where dust is produced (WHO 1999). The principle of local exhaust ventilation involves a controlled and directional airflow across an emission point and into a hood that is connected to a ductwork system. Risk has always emerged due to the increasing exposure of people who have a distance from the dust source in most ventilation systems. ACGIH (1998) provides detailed technical information about exhaust ventilation systems. Also, general ventilation could be used to control airborne dust. It helps to reduce skin and clothing contamination and dust accumulation on surfaces. For the civil engineering laboratories, local exhaust ventilation systems are suggested, considering the natural ventilation already present in the building. Quality assurance of the ventilation systems and routine checks are essential to ensuring efficient and continued performance of ventilation.

Due to the budget restrictions of the department, it is not possible to procure a local exhaust ventilation system. Therefore, measurements were repeated to examine the impact of natural ventilation. The experiments involving sieve analysis (1), preparation of concrete mixture (2), and crushing aggregate by jaw crusher (3) were measured with all windows open in the laboratories. The results of this applied control measure to reduce indoor PM levels are given in Table 5. Though the impact of natural ventilation was not as robust as the preceding substitution strategy, it remains noteworthy that the engineering control measures, although constrained by budget limitations, still contributed to a reduction in PM levels. This observation further underscores the interconnection between the experimental procedures and the control measures employed, affirming that diverse approaches could contribute to improving indoor air quality despite inherent limitations.

**Table 5:** PM concentrations of engineering control measures applied to experiments

Experiments	PM_1.0_ (µg/m^3^)			PM_2.5_ (µg/m^3^)			PM_4.0_ (µg/m^3^)			PM_10_ (µg/m^3^)		
	*min*	*max*	*mean*	*min*	*max*	*mean*	*min*	*max*	*mean*	*min*	*max*	*mean*
1	25	1901	332	11	4019	393	21	4634	753	19	9962	987
2	20	5662	215	34	2617	276	44	3097	359	23	7663	576
3	7	974	191	9	2250	378	10	2432	344	49	3673	420

The fourth step is administrative control. Training could be provided to the testers and instructors before they use laboratory facilities. The duration of the experiments could be minimized by performing the tasks more efficiently and on time. Work schedules could also be adjusted to allow short breaks for the workers to minimize PM exposure.

**Fig. 6:**
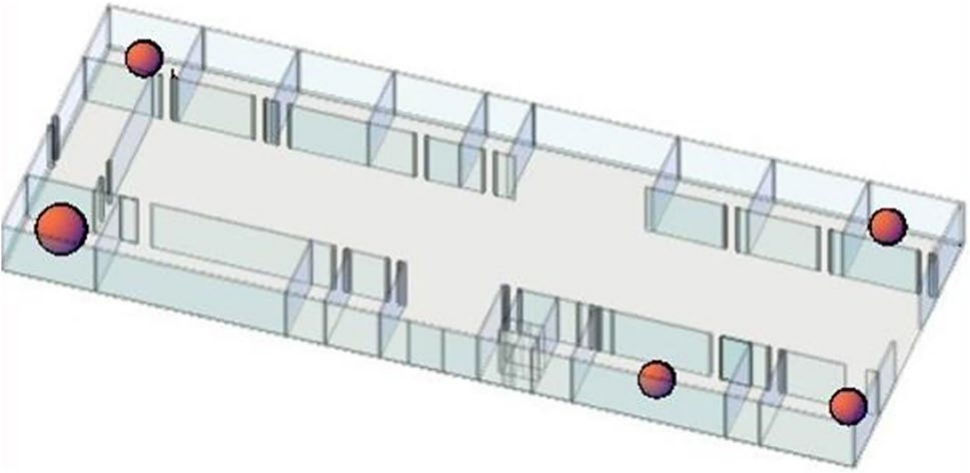
The locations of high PM levels in civil engineering laboratories

The last step is using personal protective equipment (PPE), which is the least desirable measure. All control possibilities should be explored before using the PPE, and respiratory protective equipment should be used during the experiments for temporary solutions (NIOSH 1996). The proper use of respirators has been identified in the literature (HSE 1998; OSHA 1998). In addition, an important complementary measure is good housekeeping, which helps avoid contamination spreading and unnecessary exposure. For the application of the fourth and fifth steps, training is provided to the students before each experiment by the instructors. The warning signs were placed at the entrance of the laboratories, which contain high levels of PM, to promote the usage of personal protective equipment in Fig. 6. Also, all laboratories are regularly cleaned by attendants every week.

## Conclusions

This study is structured to determine PM concentrations from experiments in civil engineering laboratories. Civil engineering laboratories are selected due to the fact that dust exposure is substantially high during educational activities. Four different PM concentrations—PM_1.0_, PM_2.5_, PM_4.0_, and PM_10_—were measured during the experiments of sieve analysis, preparation of the concrete mixture, crushing aggregate by jaw crusher, dynamic triaxial compression test, sieve analysis of the silt specimen, cleaning sieve by air compressor, and proctor compaction test, which are conducted regularly in the Civil Engineering Laboratories of Ege University, Turkey. The majority of the studies on PM measurement focused on PM_2.5_ and PM_10_ emissions. Those two sizes also underlie all the standards. However, this study included two additional PM sizes—PM_1.0_ and PM_4.0_—to gather supplementary emission data and conduct a comprehensive analysis of particle distribution within civil engineering laboratories. These measurements contributed to understanding indoor air quality during laboratory and construction processes, complementing conventional PM_2.5_ and PM_10_ assessments. Consequently, it was possible to investigate all emitted particles under coarse particles (PM_2.5–10_), fine particles (PM_2.5_), and ultra-fine particles (PM_1.0_).

The measured values are mainly high compared to the environmental IAQ standards and those of other educational laboratories. The highest levels of PM concentrations are obtained in sieve analysis in construction material laboratories, and the lowest levels are sampled in experiments in geotechnical laboratories. The average PM emissions for the experiments ranged between 83.3 and 474.3, 144.7 and 587, 180 and 972.3, and 216 and 1463 µg/m^3^ for PM_1.0_, PM_2.5_, PM_4.0_, and PM_10_, respectively.

Hierarchical implementation of hazard control is recommended to avoid health problems for both laboratory personnel and students because they are exposed to high PM concentrations during the experiments. For that purpose, the control measures were applied to the three experiments that contained the highest PM levels. The equipment for the experiments of sieve analysis, concrete mixer, and jaw crusher was replaced with safer equipment that involves closed canisters. The PM concentrations fell below the air quality standard of the EPA, changing the equipment of the experiments. The results have shown that changing the test apparatus could make a remarkable difference in protecting the health and safety of the users. Additionally, the same experiments were conducted with all windows open in the laboratories to implement the engineering control step of the hierarchy. However, while the impact of natural ventilation was not as robust as the preceding substitution strategy, it is important to emphasize that the engineering control measures played a role in decreasing PM levels.

It is expected that the results of this study will help raise awareness of construction dust hazards in construction and civil engineering education laboratories. Since civil engineering education is almost similar in most countries, the study results will also be useful for other civil engineering departments in different countries. Future research could include studying the effectiveness of other control measures for PMs, and measurements could be expanded on other experiments or activities performed in civil engineering and other engineering laboratories. Furthermore, this study revealed that there is a significant difference in PM concentrations between open construction sites and closed laboratory environments. Open construction sites, exposed to outdoor conditions and reliant on natural airflow, might exhibit lower PM levels due to activities that are influenced by weather conditions. Conversely, enclosed laboratory environments lacking applied mitigation strategies, air conditioning systems, or natural ventilation could result in higher PM levels. Future studies could analyze these differences further and explore the reasons behind concentration variations.

## Data Availability

The data that support the findings of this study are available from the corresponding author upon reasonable request.
